# Imported falciparum malaria among adults requiring intensive care: analysis of the literature

**DOI:** 10.1186/1475-2875-13-79

**Published:** 2014-03-05

**Authors:** Michael Marks, Margaret Armstrong, David Walker, Tom Doherty

**Affiliations:** 1 The Hospital for Tropical Diseases, Mortimer Market Centre, Capper Street, London WC1E 6JB, UK; 2 Department of Clinical Research, Faculty of Infectious and Tropical Diseases London School of Hygiene and Tropical Medicine, Keppel Street, London, UK; 3 Department of Critical Care, University College London Hospital, London, UK

**Keywords:** Malaria, Imported infections, ICU, ARDS

## Abstract

**Background:**

Malaria is the most important imported tropical disease. Infection with *Plasmodium falciparum* is responsible for most of the morbidity and mortality. There are differences in both the epidemiology of imported malaria and in the facilities available to treat travellers with severe malaria between different parts of the world. There are limited data to guide clinicians caring for adults with imported malaria in an intensive care unit (ICU). Available data from the English-speaking literature concerning such patients was reviewed.

**Methods:**

PubMed was searched for studies on adults with imported malaria treated in an ICU. Data were extracted on the epidemiology, management, rates of concomitant community-acquired bacterial infection and outcomes.

**Results:**

Thirteen studies were identified, which between them included 1,001 patients over more than 40 years. Forty-one per cent were born and often still resident in an endemic country and were assumed to have at least partial immunity to the disease. Acute kidney injury (AKI) (36%), acute respiratory distress syndrome (ARDS) (31%) and impaired consciousness (25%) were common. Hyperparasitaemia (more than 2%) was seen in 57%. Thirty-four per cent required mechanical ventilation and 22% required renal replacement therapy. Community-acquired bacterial co-infection was seen in 8%; 2% had gram-negative bacteraemia at admission. Overall the case fatality rate was 9%.

**Conclusions:**

Many patients who require admission to ICU were originally from malaria-endemic countries and many did not have hyperparasitaemia. Gram-negative bacteraemia was uncommon among adults with severe malaria. The case fatality rate remains high; however, improvements in ICU care and increasing use of artemisinins may reduce this in the future.

## Background

Malaria is the most important imported tropical disease and responsible for most deaths that occur among individuals returning from the tropics. *Plasmodium falciparum* is responsible for virtually all cases of imported malaria that require admission to an intensive care unit (ICU), including those that die, which may be as many as 29% [1].

Most descriptions of the clinical spectrum of severe malaria disease come from countries where the disease is endemic and predominantly affects young children. In these settings, adults are assumed to develop some degree of immunity to the disease, although this is rapidly lost if they move away from an endemic area [2]. The precise mechanisms that contribute to this acquired immunity remain poorly understood [2] and there is no agreed definition of ‘immunity’ in studies of imported malaria. Relatively few data have been published concerning the pattern of disease among adult patients with severe imported malaria. The aim of this study was to review all published series of falciparum malaria requiring admission to an ICU in resource-rich countries to determine the reasons for admission, complication and case fatality rates and the frequency of concomitant bacterial sepsis, which has been reported to be very high among African children with the disease [3].

## Methods

### Search method

PubMed was searched for any papers published. Combinations of the following search terms were used: “malaria”, “falciparum”, “ITU”, “ICU”, “intensive care”, “imported” and “non-endemic”. The abstracts of all these papers were reviewed for eligibility by MM. The bibliographies of eligible articles were screened to identify any other relevant publications.

### Study selection

Papers were included only if they reported data on the care and outcome of adult (aged ≥18 years) patients with imported malaria requiring admission to ICU. For pragmatic reasons case series of fewer than ten patients were excluded.

### Data extraction

Data from each paper were extracted on to a standardized form. Where possible, data were recorded about demographics, region of travel, use of anti-malarial chemoprophylaxis, the management of malaria, including requirements for organ support, complications of malaria, rates and nature of bacterial co-infection and outcome.

### Statistical analyses

Continuous variables were described with mean and standard deviation (SD) or with median and interquartile range (IQR) as appropriate. Categorical data were described with numbers and per cent. All analyses were carried out using Stata 10 (Statacorp).

## Results

### Literature search

**Figure 1:**
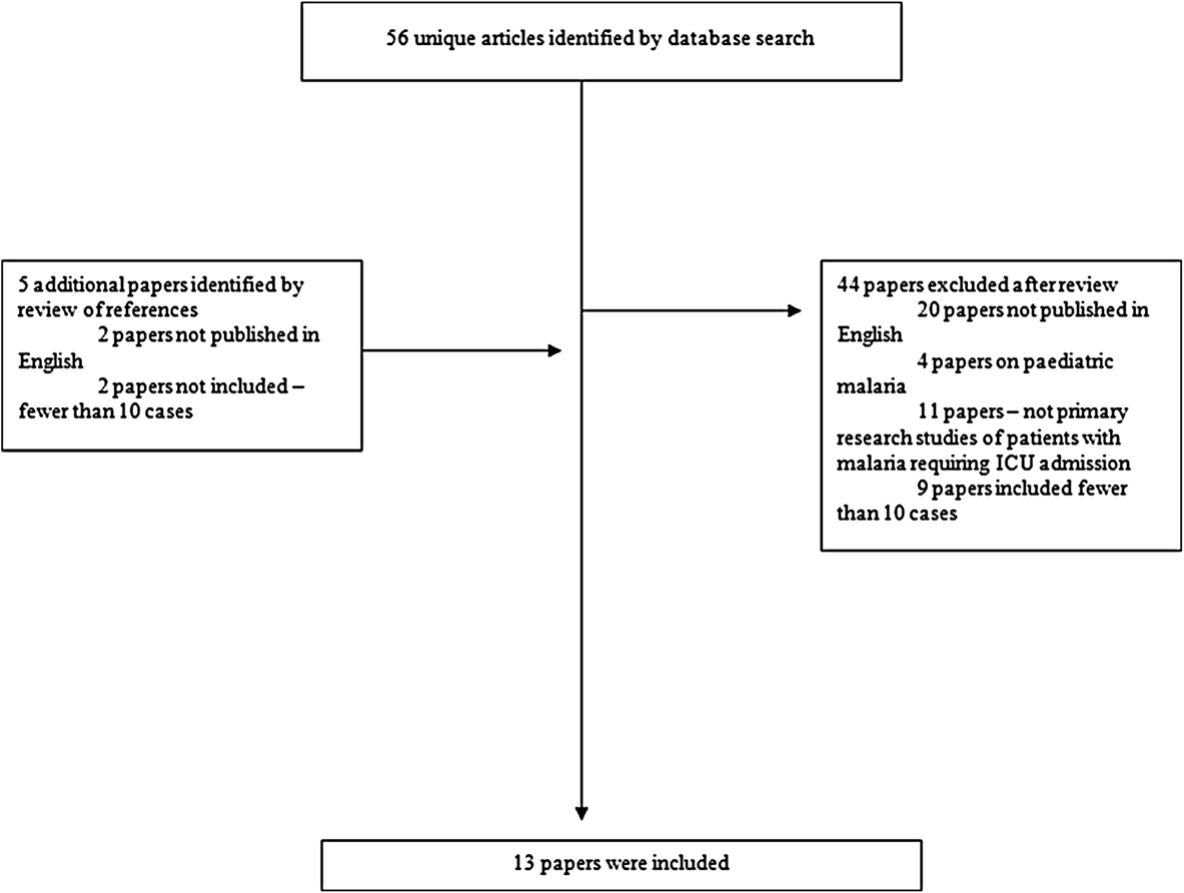
Search strategy.

The literature search identified 61 papers, 13 of which were included (Figure 1) [1,4-15]. Forty-eight were excluded; 22 were not published in English, 11 were not primary research articles, four reported only on paediatric cases and 11 were short case series that included fewer than ten patients. The 13 studies reviewed included a total of 1,001 patients over a 41-year period (Table 1). All 13 were retrospective cohort studies; 11 reported patients treated in Europe; the other two were from Singapore and South Africa. Four studies included some patients with severe malaria that were not admitted to an ICU and seven included some aged under 18 years (n = 15 in total); where possible, individual patient data were used to exclude these from further analyses. Where it was not possible to exclude paediatric patients from the analysis, this is made that clear.

**Table 1:** Included studies

**Study**	**Country**	**Time period**	**Study type**	**Number of patients**
Badiaga [4]	France	1996-2002	Single centre	37^a^
Blumberg [1]	South Africa	1993-1994	Single centre	23^b^
Bruneel 2003 [5]	France	1988-1999	Single centre	188
Bruneel 2010 [6]	France	2000-2006	Multi centre (n = 45)	400
Gachot [7]	France	1988-1993	Single centre	40
Gonzalez [8]	Spain	1991-2007	Single centre	20^c^
Khoo [9]	Singapore	1994-1997	Single centre	19^d^
Marks [10]	United Kingdom	1994-2010	Single centre	124
Nüesch [11]	Switzerland	1970-1992	Multi centre (n = 2)	23
Salord [12]	France	1985-1990	Single centre	19^e^
Santos [13]	Spain	1990-2011	Single centre	56^f^
Schwake [14]	Germany	1996-2003	Single centre	13
Thierfelder [15]	Switzerland	1994-2004	Single centre	38^g^

^a^Two paediatric cases excluded.

^b^Five paediatric cases excluded where possible.

^c^At least one patient was aged under 18.

^d^One paediatric case excluded.

^e^Two paediatric cases excluded from further analyses where possible.

^f^Three paediatric cases excluded from further analyses where possible.

^g^At least one patient was aged under 18 and one patient had Vivax infection: Individual patient data were not available therefore these patients could not be excluded from further analyses.

### Epidemiology

Of the 1,001 patients, men aged between 30 and 50 made up the largest group (Table 2). In studies in which ethnicity was reported (nine studies, 916 patients), most were originally from non-endemic countries (59%, range 49-100%). Except for the paper from Singapore [9], *P. falciparum* was almost always acquired during travel to sub-Saharan Africa. Use of appropriate anti-malarial chemoprophylaxis was poor (<20%) in all studies.

**Table 2:** Demographics and epidemiology

**Study**	**Male**	**Median age (Median + IQR, or Mean ± SD)**	**Semi immune**^**a**^	**Acquired in Africa**	**Adequate chemoprophylaxis**
Badiaga	73%	45 (IQR 29-55)	17%	100%	5%
Blumberg	61%	38 (IQR 30-48 · 5)	Not reported	100%	Not reported
Bruneel 2003 [5]	63%	38 (no IQR available, range 14-74),	49%	94%	4%
Bruneel 2010 [6]	70%	42 · 8 ± 15^b^ + 55 · 6 ± 14 · 2^c^	40%	96%	16%
Gachot	58%	39 · 2 ± 3 · 7^d^ + 38 · 3 ± 0 · 2^e^	35%	95%	Not reported
Gonzalez	75%	44 (No IQR available, range 16-68)	0%	95%	0%
Khoo	89%	38 (IQR 29 · 5 – 50 · 5)	Not reported	21%	0%
Marks	63%	46 (IQR 35-55)	45%	94%	1%
Nüesch	83%	42 (IQR 37 · 5-51)	Not reported	Not reported	9%
Salord	74%	37 (IQR 28 · 5-42 · 5)	Not reported	95%	0%
Santos	80%	42 (IQR 33-50)	51%	95%	2%
Schwake	72%	38 · 4 ± 11 · 4^f^	28%	88%	20%
Thierfelder	50%	38 (no IQR available, range 16-71)^g^	49%	82%	Not reported

^a^Definitions of Semi-Immune provided in Additional file 1.

^b^Survivors ^c^Non-Survivors.

^d^Patients with Acute Lung Injury ^e^Patients without Acute Lung Injury.

^f^Not all patients in the study were admitted to ICU - Demographic data for only those patients admitted to ICU was not available.

^g^Not all patients in the study were admitted to ICU - Demographic data for only those patients admitted to ICU was not available. Individual patient data were not available therefore it was not possible to exclude the paediatric cases from these figures.

The definition of “immunity” to malaria varied between studies (Additional file 1). Individual patient data was not available to allow a consistent definition of immunity to be applied across all studies.

### Treatment of malaria and ICU care

Quinine was the usual first-line drug in all 13 studies (Table 3). Use of artemisinins was reported in only one paper [10]. In the study by Nüesch [11], chloroquine with or without sulphadoxine/pyrimethamine was the first-line anti-malarial treatment before 1987. In those studies limited exclusively to patients admitted to ICU, the proportion of patients requiring renal replacement therapy, mechanical ventilation and inotropic support were 22% (95% CI 19-25%), 34% (95% CI 32-38%) and 27% (95% CI 24-30%), respectively. Only two small studies (n = 52) provided adequate detail to assess requirements of individual patients for multiple organ support. In these two studies 44% of patients required at least two organ support. Six studies reported the use of exchange transfusion in a median of 25% of patients (IQR 18-29%). The median length of stay on ICU ranged from –four to 11 days.

**Table 3:** Management of patients

**Study**	**Quinine**	**Artemisinins**	**Renal replacement therapy**	**Invasive ventilation**	**Inotropic support**	**Exchange transfusion**	**Length of ICU admission (Median days, IQR)**
Badiaga	100%	0%	Not reported	Not reported	Not reported	Not reported	4 (2-14)
Blumberg	100%	0%	48%	57%	57%	17%	8 (5-13)
Bruneel 2003 [5]	100%	0%	15%	43%	13%	0%	8 (4-13) 2 (1-4)
Bruneel 2010 [6]	98%	Not reported	20%	29%	27%	0%	4 (2-7) 5 (3-8)
Gachot	100%	0%	Not reported	23%	Not reported	Not reported	11 (No IQR available)
Gonzalez	100%	0%	40%	50%	Not reported	30%	5 (3-12)
Khoo	100%	0%	47%	42%	37%	32%	5 +/- 4(Mean)
Marks	99%	17%	35%	37%	35%	27%	10 (7-9)
Nüesch	15% (Pre-1987) 100% (1987-)	0%	9%	26%	22%	22%	6 (2-28)
Salord	89%	0%	5%	42%	11%	16%	Not reported
Santos	100%	0%	14%	37%	49%	Not reported	4 (2-12) 8 (3-27)
Schwake	100%	0%	7%	5%	8%	Not reported	5 (3-6)
Thierfelder	Not reported	Not reported	Not reported	Not reported	Not reported	Not reported	Not reported

### Bacterial co-infections

The rate of community-acquired bacterial co-infection was reported in seven studies (n = 855, including four paediatric cases) with an overall prevalence of 8% (n = 66; range 0-13%). Pneumonia was the most common community-acquired co-infection occurring in 28 (3%) cases. Bacteraemia, defined as a positive blood culture within 48 hours of admission, was found in 25 (3%) cases; most of these (18, (72%)) were caused by gram-negative bacteria.

### Complications of *Plasmodium falciparum* infection

Definitions of complications, including hyperparasitaemia, varied between studies (Additional file 2). The frequency with which these complications occurred is shown in Table 4.

**Table 4:** Complications

**Study**	**Hyper-Parasitaemia**	**Acidosis**	**AKI**	**Impaired consciousness**	**Shock**	**Coagulopathy**	**ARDS**	**Hypoglycaemia**	**Jaundice**	**Seizures**	**Anaemia**	**Haema globinuria**	**Bacterial Co-Infection**	**Mortality**
Badiaga	78%	8%	35%	24%	19%	8%	24%	0%	62%	11%	0%	14%	Not reported	8%
Blumberg	79%	36%	61%	39%	46%	14%	46%	11%	39%	11%	21%	0%	Not reported	29%
Bruneel 2003 [5]	21%	10%	25%	18%	11%	12%	5%	2%	27%	1%	8%	1%	10%	5%
Bruneel 2010 [6]	64%	18%	34%	26%	24%	3%	25%	3%	52%	7%	4%	6%	8%	11%
Gachot	Not reported	Not reported	55%	38%	25%	35%	30%	10%	Not reported	Not reported	8%	Not reported	13%	13%
Gonzalez	60%	30%	35%	20%	25%	55%	10%	15%	90%	10%	0%	5%	0%	25%
Khoo	45%	35%	45%	0%	30%	30%	5%	0%	75%	15%	20%	Not reported	Not reported	15%
Marks	66%	55%	44%	35%	27%	11%	19%	4%	69%	7%	4%	0%	5%	4%
Nüesch	65%	Not reported	26%	17%	9%	4%	4%	Not reported	4%	Not reported	13%	Not reported	13%	17%
Salord	58%	Not reported	32%	47%	Not reported	Not reported	26%	Not reported	42%	16%	Not reported	Not reported	Not reported	0%
Santos	78%	39%	53%	19%	49%	22%	37%	31%	46%	Not reported	0%	Not reported	3%	15%
Schwake	Not reported	31%	38%	23%	38%	31%	15%	23%	Not reported	0%	0%	Not reported	Not reported	0%
Thierfelder	Not reported	Not reported	Not reported	Not reported	Not reported	Not reported	Not reported	Not reported	Not reported	Not reported	Not reported	Not reported	Not reported	0%

Ten studies (n = 918, including 14 paediatric cases) reported the presence of hyperparasitaemia, which was seen in 523 (57%) patients, using a cut-off of 2% (95% CI 54-60%). When only studies using a cut-off of >5% were analysed, the prevalence of hyperparasitaemia was 44% (95% CI 39-49%).

Twelve studies (n = 971, including 14 paediatric cases) reported the presence of AKI, which was seen in 350 patients (36%) (95% CI 33-39%). The proportion of patients with acute kidney injury (AKI) did not differ significantly between studies that defined this as both oliguria and elevated creatinine and those studies that used only an elevated creatinine (40 *vs* 34%, p = 0 · 1280) to define AKI.

Twelve studies (n = 971, including 14 paediatric cases) reported the presence of acute respiratory distress syndrome (ARDS), which was seen in 301 patients (31%; 95% CI 28-34%). Two studies (n = 161) [6,10] reported both complications at admission and complications that developed during the ICU stay; ARDS was present at admission in three (2%) and developed later in a further 32 (20%).

Ten studies (n = 952, including 12 paediatric cases) reported the presence of impaired consciousness, used as a proxy for cerebral malaria, which was seen in 238 patients overall (25%; 95% CI 22-28%).

Five studies (n = 624) reported the total number of complications that occurred per patient. The mean number of complications seen was 2.86% (95% CI 2.81-2.91%). Individual patient data was not available to assess if particular combinations of complications occurred with an increased frequency.

WHO defines severe malaria using a range of other criteria that include: acidosis, coagulopathy, hypoglycaemia, jaundice, and anaemia. The rates of these complications, where available, are shown in Table 4.

Case fatality rates were reported in all 13 studies. The overall case fatality rate was 9% (95% CI 8-11%) but a case fatality rate of over 15% was reported in four studies.

## Conclusions

This is the first review of imported falciparum malaria among adults who were sufficiently unwell to require admission to ICU. The review supports four important conclusions. First, hyperparasitaemia, however this is defined, is not a feature in almost 50% of patients; second, up to 40% of these patients were originally from or still resident in a malaria-endemic region and may therefore be assumed to have at least partial immunity to the disease; third, co-infection with community-acquired gram-negative bacteraemia is uncommon among adults; and, finally, the case fatality rate for severe imported malaria in ICU is in the region of 10%.

AKI, ARDS and cerebral malaria were all common. Invasive ventilation was required in 22% and renal replacement therapy was required in approximately 22%. Hyperparasitaemia, which is often used as a proxy for severe falciparum, was seen in only 57% of subjects when a cut-off of ≥2% was used. When using a definition of ≥5% parasitaemia, which the WHO recommends as the criterion for severe disease in endemic areas, this proportion fell to 44%. Clinicians would be well advised not to rely on parasite count as the only indicator of severe disease.

Gram-negative bacteraemia, classically with non-typhoidal *Salmonellae*, is a well recognized complication of malaria in children [3], occurring in up to 20%. Recent studies have suggested that dysfunctional granulocyte mobilization may be responsible for this [16]. While 8% of patients had a presumed community-acquired bacterial co-infection, usually pneumonia, at admission, only 25 (3%) had culture-positive, gram-negative bacteraemia. Previous prescription of antibiotics may have caused some bias and a lower yield but it is possible that this reflects a difference in the pathophysiology of severe malaria in adults compared to children.

The overall case-fatality rate was 9% although one study [1] reported a rate as high as 29%. Quinine was the most commonly used anti-malarial agent in all 13 studies. Artesunate has been shown to significantly reduce case fatality from severe malaria in both Southeast Asia and Africa [17,18]. Only one of these studies [10] included patients treated with artemisinins, but as these drugs become more widely available the case fatality from severe imported malaria may decline.

This review has several weaknesses. A number of studies that were not published in English were excluded and it is possible that this may have had an effect. Most of the included studies were from Europe and the conclusions may not be relevant to other settings where imported malaria is seen. In particular, indications for ICU admission are likely to have varied both between the study sites and over the period that the studies were conducted, which may partially explain the spectrum of disease severity and outcomes seen. Individual patient data were often unavailable, which meant paediatric cases could not reliably be excluded, although there were only 15 of these. For the same reasons, it was not possible to analyse temporal trends in case fatality rates or perform a detailed meta-analysis of risk factors associated with mortality. However, the review includes all series of more than ten patients published in English over more than 40 years and provides important insights into the management of this relatively uncommon disease.

It is noteworthy that no case series from North America were included in the current paper. In 2011 the United States reported 1,925 cases of America of which 183 were reported to have severe disease [19]. Given the significantly larger population of the United States, compared to the other countries represented in this paper, it is possible that no American centre sees a sufficiently large enough number of cases to inform a study of severe malaria in the USA.

The overall case fatality rate of 9% may not reflect modern practice. Management of ARDS has improved considerably in recent years and newer strategies for fluid resuscitation, in particular, may reduce the incidence of both multi-organ failure and long-term disability [20]. Similarly, optimizing fluid management for patients with ARDS may reduce pulmonary capillary leak and subsequently the length of time that patients require on ICU [21]. Greater awareness of what constitutes AKI [22] may also result in a better outcome. Each of these factors, as well as improved antimicrobial protocols, management of shock and acidosis and increasing use of artemisinins, may further reduce the case fatality associated with severe imported malaria in the future.

## Competing interests

The authors declare that they have no competing interests.

## Authors’ contributions

MM undertook the literature search, performed the data collection and analysis and wrote the first draft of the paper. MA contributed to data interpretation, figures and writing of subsequent drafts. DW contributed to data interpretation and writing of subsequent drafts. TD initiated the project, contributed to data interpretation and the writing of subsequent drafts. All authors read and approved the final manuscript.

## Supplementary Material

Additional file 1 **Definitions of Immunity.** The data provided outline the definitions of ‘immunity’ used in the studies included in this paper. Click here for file

Additional file 2 **Definitions of Complications.** The data provided outline the definitions of complications of malaria used in the studies included in this paper. Click here for file
