# Supplementary material for: Imported falciparum malaria among adults requiring intensive care: analysis of the literature
Source: Malar J. 2014 Mar 5;13:79. doi: 10.1186/1475-2875-13-79 (PMC4015364; doi:10.1186/1475-2875-13-79)
Supplement: Additional file 1 — Definitions of Immunity. The data provided outline the definitions of ‘immunity’ used in the studies included in this paper. [file 1475-2875-13-79-S1.docx]

Additional File 1: Definitions of Immunity

Most studies defined semi-immune patients as individuals who had been living in an endemic country for a minimum two years at the time of diagnosis or immigrants from endemic regions who now lived in a non-endemic country.

One study divided patients into partially immune (immigrants from endemic countries) and semi-immune (individuals who had been living in an endemic country for at least two years).

One other study defined patients as being ‘malaria naive’ (born and living in a non-endemic country), “little” immunity (individuals who had been born in an endemic country but emigrated to a non-endemic country in the last two years), and “some” immunity (individuals who were born and still living in endemic regions).
