# Supplementary material for: Imported falciparum malaria among adults requiring intensive care: analysis of the literature
Source: Malar J. 2014 Mar 5;13:79. doi: 10.1186/1475-2875-13-79 (PMC4015364; doi:10.1186/1475-2875-13-79)
Supplement: Additional file 2 — Definitions of Complications. The data provided outline the definitions of complications of malaria used in the studies included in this paper. [file 1475-2875-13-79-S2.docx]

Additional File 2: Definitions of Complications

The definition of hyperparasitaemia varied between the studies; cut-off values of >2%,^12^ ≥4%,^4,5^ and >5%^1,6–10,15^ were used.

Acute kidney injury was defined as a serum creatinine >265µmols and oliguria (<400ml/24hr) in six studies.^4,8–10,12,13^ Three studies used only a creatinine >265 µmols.^6,7,15^ One study used either creatinine>265 µmols or a urea >17mm/L.^15^ One early study used a cut-off of creatinine >250 µmols.^1^

ARDS was defined as bilateral pulmonary infiltrates and PaO_2_:FiO_2_ ratio of <26·7, not attributed to left ventricular dysfunction in the opinion of the ICU clinician in all except one study^5^ in which a requirement for non-invasive and/or endotracheal mechanical ventilation or spontaneous breathing with PaO_2_ <60 mm Hg (if FiO_2_ >0·21) , and/or respiratory rate >32 breaths/minute was used.

Acidosis was defined as a pH <7·25 or a bicarbonate <15mmol/L in five studies.^6,8,9,12,13^ Three used a pH of 7·35 or a bicarbonate <15 mmol/L.^5,10,15^ One used only a pH <7·35.^1^ One used any of a pH <7·25, a bicarbonate <15 mmol/L or a lactate >15.^4^ One used only a bicarbonate <15 mmol/L.^7^

Shock was defined as either a systolic blood pressure less than <70mmHg,^9,12,13^ <80mmHg,^4–6,8,15^ <90mmHg,^7^ or a requirement for inotropic support.^1^

Unrousable coma was defined as a Glasgow Coma Score (GCS) <9,^7,13^ <10, ^4,6,8,10,12^ <11.^5,15^ Two papers^1,9^ did not define what they meant by cerebral malaria.
